# Refined analyses suggest that recombination is a minor source of genomic diversity in *Pseudomonas aeruginosa* chronic cystic fibrosis infections

**DOI:** 10.1099/mgen.0.000051

**Published:** 2016-03-02

**Authors:** David Williams, Steve Paterson, Michael A. Brockhurst, Craig Winstanley

**Affiliations:** ^1^​ Institute of Integrative Biology, Biosciences Building, University of Liverpool, Crown Street, Liverpool L69 7ZB, UK; ^2^​ Department of Biology, University of York, Wentworth Way, York YO10 5DD, UK; ^3^​ Clinical Infection, Microbiology & Immunology, Institute of Infection & Global Health, University of Liverpool, 8 West Derby Street, Liverpool L69 7BE, UK

**Keywords:** BAGA, cystic fibrosis, genomic diversity, *Pseudomonas aeruginosa*, recombination

## Abstract

Chronic bacterial airway infections in people with cystic fibrosis (CF) are often caused by *Pseudomonas aeruginosa*, typically showing high phenotypic diversity amongst co-isolates from the same sputum sample. Whilst adaptive evolution during chronic infections has been reported, the genetic mechanisms underlying the observed rapid within-population diversification are not well understood. Two recent conflicting reports described very high and low rates of homologous recombination in two closely related *P. aeruginosa* populations from the lungs of different chronically infected CF patients. To investigate the underlying cause of these contrasting observations, we combined the short read datasets from both studies and applied a new comparative analysis. We inferred low rates of recombination in both populations. The discrepancy in the findings of the two previous studies can be explained by differences in the application of variant calling techniques. Two novel algorithms were developed that filter false-positive variants. The first algorithm filters variants on the basis of ambiguity within duplications in the reference genome. The second omits probable false-positive variants at regions of non-homology between reference and sample caused by structural rearrangements. As gains and losses of prophage or genomic islands are frequent causes of chromosomal rearrangements within microbial populations, this filter has broad appeal for mitigating false-positive variant calls. Both algorithms are available in a Python package.

## Data Summary

1. Short read data for Nottingham *Pseudomonas aeruginosa* isolates were obtained from the European Nucleotide Archive; study: ERP005188 (http://www.ebi.ac.uk/ena/data/view/ERP005188).
2. Short read data for Liverpool *Pseudomonas aeruginosa* isolates were obtained from the European Nucleotide Archive; study: ERP006191; sample group: ERG001740; reads: ERR953477–ERR953516 (http://www.ebi.ac.uk/ena/data/view/ERP006191).
3. Complete genome sequence with annotations for *Pseudomonas aeruginosa* LESB58 was obtained from NCBI RefSeq: NC_011770.1 (http://www.ncbi.nlm.nih.gov/nuccore/NC_011770.1).
4. Complete genome sequence with annotations for *Pseudomonas aeruginosa* LESlike7 was obtained from NCBI RefSeq: NZ_CP006981.1 (http://www.ncbi.nlm.nih.gov/nuccore/NZ_CP006981.1).
5. The Python package ‘Bacterial and Archaeal Genome Analyser’ (BAGA) can be used to download the data, and reproduce most of the analysis, tables and figures. The most recent version is available from the GitHub repository: https://github.com/daveuu/baga; release version 0.2: http://dx.doi.org/10.6084/m9.figshare.2056350
6. A script to reproduce the analysis using BAGA is available via FigShare: http://dx.doi.org/10.6084/m9.figshare.2056359
7. A script to reproduce the benchmarking of variant calling using BAGA is available via FigShare: http://dx.doi.org/10.6084/m9.figshare.2056365
8. Variants called against the *Pseudomonas aeruginosa* LESB58 and LESlike7 genomes and for benchmarking are available as VCF files via FigShare: http://dx.doi.org/10.6084/m9.figshare.2056326
9. Variants called against the *Pseudomonas aeruginosa* LESB58 and LESlike7 genomes and for benchmarking are available as CSV files via FigShare: http://dx.doi.org/10.6084/m9.figshare.2056356
10. The multiple sequence alignments from which the phylogeny and recombination were inferred are available via FigShare: http://dx.doi.org/10.6084/m9.figshare.2056344

## Impact Statement

Rapid pathogen evolution within chronic infections is a major health concern. The resulting high levels of genetic diversity within patients can make infections harder to diagnose and treat. Understanding the genetic mechanisms by which this genetic diversity is generated is therefore vitally important. Two recent studies using genomics to analyse populations of *Pseudomonas aeruginosa* causing chronic airway infections in cystic fibrosis patients reported conflicting findings. Estimates of the contribution of genetic exchange by homologous recombination, a process that could potentially accelerate pathogen adaptive evolution by generating diversity, differed between the two reports. We applied a new analytical approach to the genome data from these studies that, by inclusion of a stringent data-filtering regime, was designed to improve accuracy. In both sets of data, we found similarly low rates of genetic exchange. This suggests that *de novo* mutation, not genetic exchange, is the primary mechanism driving evolutionary diversification of bacterial populations in these chronic infections.

## Introduction

People with cystic fibrosis (CF) are susceptible to a range of bacterial airway infections, most commonly due to *Pseudomonas aeruginosa*, which once established are difficult to clear. Damage to lung tissue caused by these chronic infections is a major cause of patient morbidity and mortality. A number of studies have used analyses of sequentially sampled single-isolate genome sequences obtained over many years from CF patient sputum to characterize adaptive evolution during chronic infection (Smith *et al.*, 2006; Cramer *et al.*, 2011; Marvig *et al.*, 2013). However, recent studies have demonstrated that there is considerable phenotypic and genomic diversity within single populations of *P. aeruginosa* in the CF lung (Mowat *et al.*, 2011; Workentine *et al.*, 2013; Williams *et al.*, 2015). Darch *et al.* (2015) reported large trade-offs in virulence factors, quorum sensing signals and growth amongst CF lung *P. aeruginosa*. Notably, they discovered that when multiple isolates were mixed together, resistance to antibiotics increased significantly. As this diversity impedes accurate diagnosis and treatment, there is an urgent need to understand the mechanisms by which these complex population structures have evolved.

Although most patients become infected with a *P. aeruginosa* strain from the environment, transmissible strains can lead to cross-infection between CF patients (Winstanley *et al.*, 2009; Fothergill *et al.*, 2012). In the UK, the most abundant single lineage associated with chronic lung infections with CF patients is the Liverpool Epidemic Strain (LES) (Fothergill *et al.*, 2012; Martin *et al.*, 2013). Recent reports by Darch *et al.* (2015) and Williams *et al.* (2015) estimated the amount of genetic exchange by homologous recombination in populations of the *P. aeruginosa* LES from chronic infections of CF airways. Both studies sequenced genomes of multiple contemporary isolates from individual patient sputum samples, but whereas Darch *et al.* (2015) inferred high rates of recombination correlated with phenotypic diversity, Williams *et al.* (2015) reported much lower rates, implying a larger role for spontaneous mutations in generating diversity.

In this study, we describe a novel and easily reproducible analysis of whole-genome short reads from the Darch *et al.* (2015) and Williams *et al.* (2015) papers to estimate recombination rates amongst *P. aeruginosa* LES populations during chronic infection of the airways of two CF patients. We conclude that differences in the bioinformatic analyses can explain the contradictory findings between the two studies and that although recombination occurs, it is not the major driver of the population heterogeneity observed amongst infecting populations of *P. aeruginosa* in these patients.

## Methods

The whole variant calling bioinformatic analysis pipeline can be conveniently reproduced using the freely available ‘Bacterial and Archaeal Genome Analyser’ (BAGA) command line tool and Python 2.7 package, tested on Linux. See Data Bibliography for commands to reproduce the analysis and benchmarking. Each set of short reads was aligned to two reference genomes: *P. aeruginosa* LESB58 (Winstanley *et al.*, 2009) and LESlike7 (Jeukens *et al.*, 2014). Variants were called using the Genome Analysis Toolkit (GATK) HaploTypeCaller (McKenna *et al.*, 2010; DePristo *et al.*, 2011; Van der Auwera *et al.*, 2013). Two novel variant filters were developed to mitigate false-positive variants at regions likely to be affected by rearrangements between a sample and the reference and repeat regions in the reference. Variants were validated by confirming their existence in contigs generated by *de novo* assembly of the small subset of reads aligning to regions around variants using SPAdes (Bankevich *et al.*, 2012). The accuracy of the pipeline was benchmarked using simulated reads containing known variants using GemSIM (McElroy *et al.*, 2012). See online Supplementary Material for further details.

## Results and Discussion

### Both populations exhibit similarly low rates of recombination

**Fig. 1.**
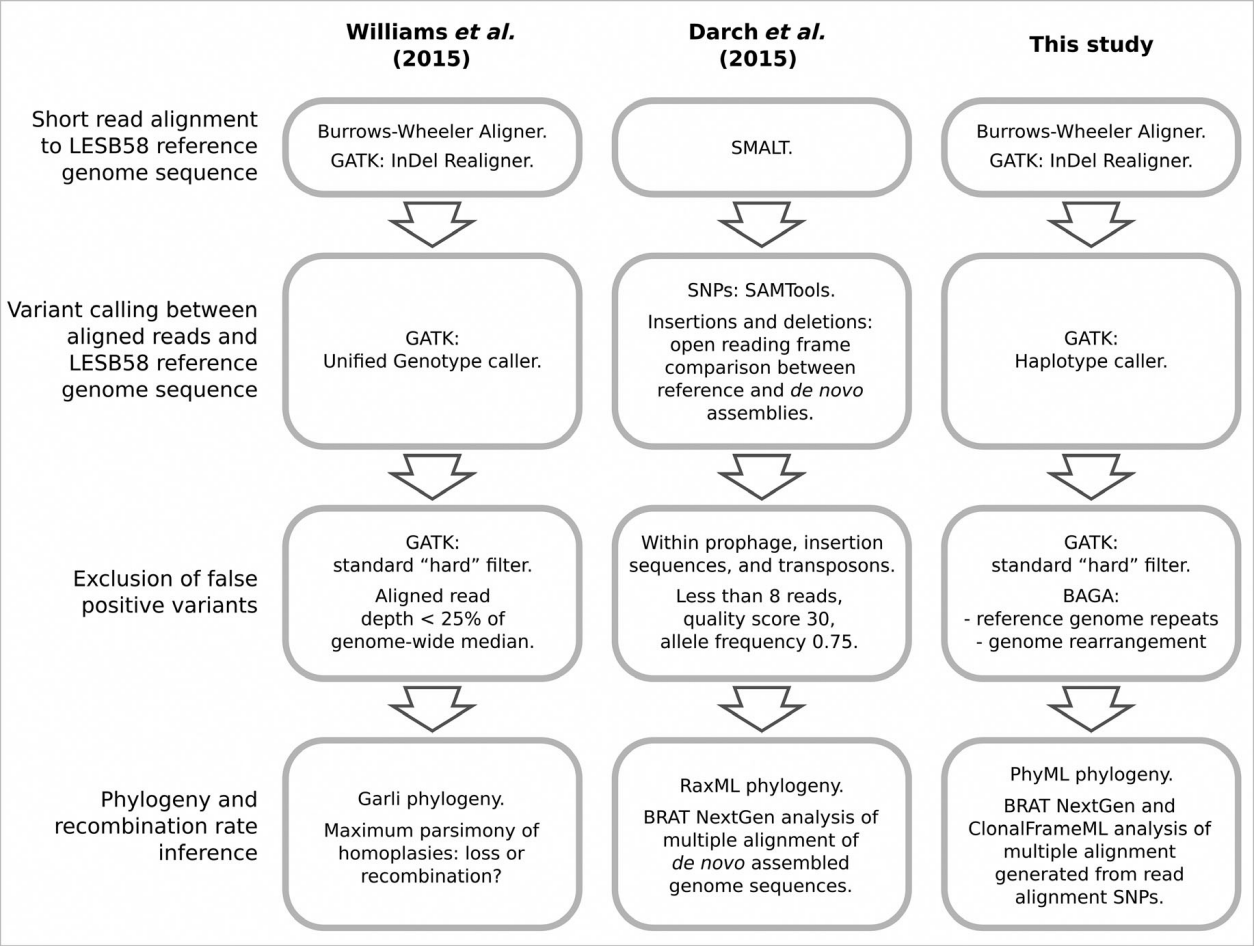
Comparison of stages of bioinformatic analyses in this and the two previous studies (Darch *et al.*, 2015; Williams *et al.*, 2015). The relevant aim of each analysis was estimation of recombination rates amongst *P. aeruginosa* isolates from short read whole-genome shotgun sequencing data.

This analysis incorporates genomic short read Illumina data from two previous studies. All short read data from the Darch *et al.* (2015) report were included, representing 22 of the *P. aeruginosa* isolates obtained from a single sputum sample from a chronically infected CF patient at a Nottingham clinic. These will be referred to as the Nottingham data. A subset of the short read data from the Williams *et al.* (2015) report, that sequenced from 40 *P. aeruginosa* isolates obtained from the patient ‘CF03’ sputum sample, were incorporated and will be referred to as the Liverpool data. Differences in the methods of the two previous papers are summarized in Fig. 1.

**Fig. 2.**
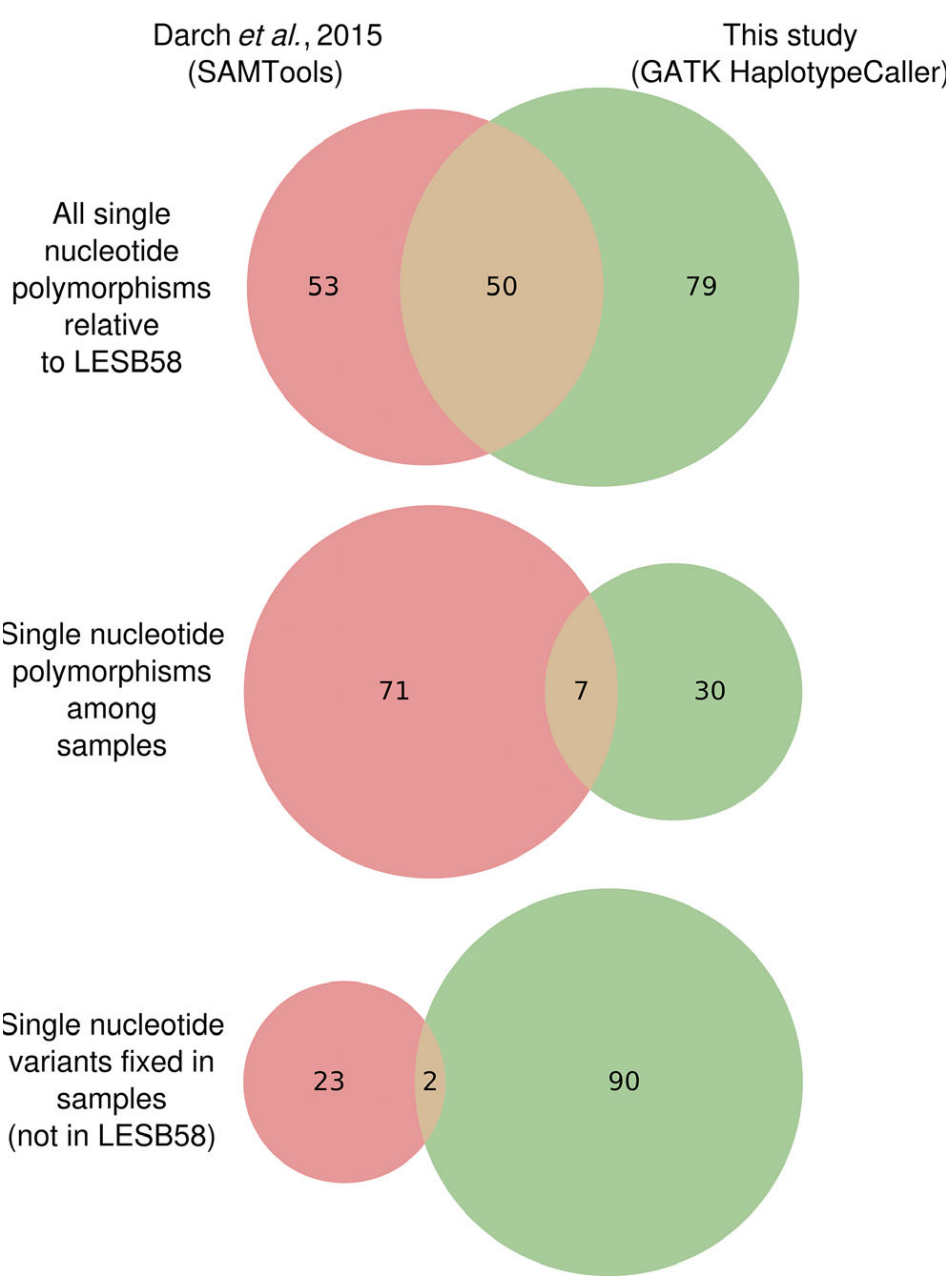
Congruity between two independent variant calling analyses on the same dataset.

**Fig. 3.**
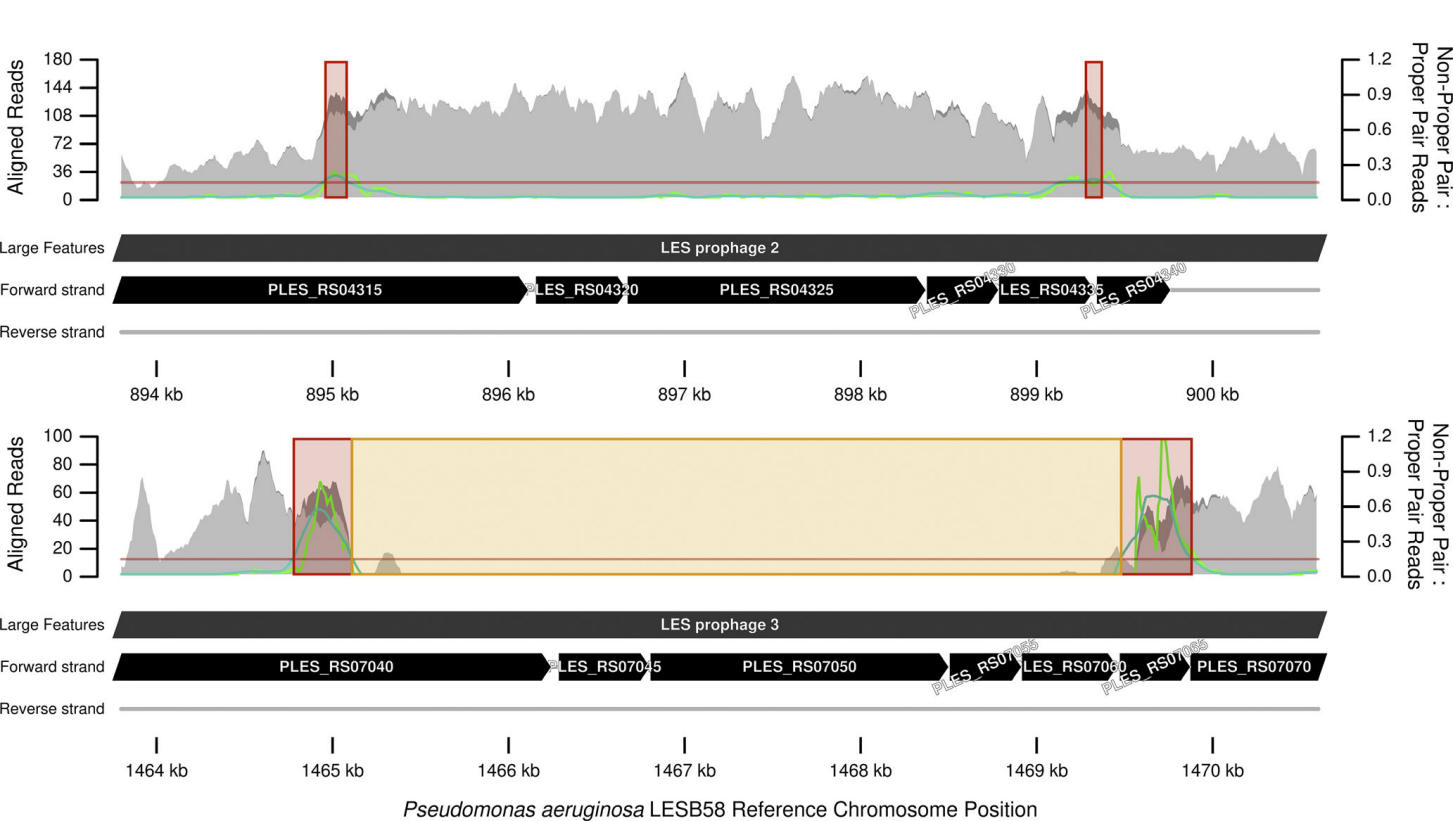
Sequence repeat units in *P. aeruginosa* LESB58 that have disrupted paired short read alignments for a closely related isolate. Light grey is depth of aligned reads designated in a ‘proper pair’ by the aligner algorithm; dark grey, stacked above light grey, is depth of reads not in a proper pair. Light green is the ratio of reads not in a proper pair to those in a proper pair; dark green is this ratio ‘smoothed’ by averaging over a 500 bp moving window. Read mapping for variant calling assumes a one-to-one orthologous relationship between sample and reference genome. The proportion of proper pair aligned reads decreases where this orthologous relationship breaks down at areas of chromosomal rearrangement or when the orthology becomes ambiguous, such as repeat regions. Variants called within a zone where the smoothed average ratio exceeds a threshold of 0.15, highlighted in red, have a higher chance of being false positives and are omitted. Variants are also omitted from neighbouring zones with discontinuous regions of aligned reads, highlighted in orange. Several variants were filtered at this region. This region was also detected by the ‘repeats’ filter.

In the present analysis, 270 SNPs and 60 indels were called between the Liverpool sequences and the LESB58 reference sequence compared with 251 SNPs and 49 indels reported previously (Williams *et al.*, 2015). For the Nottingham sequences, 129 SNPs were called. This is similar in quantity to a set of 121 SNPs reported by Darch *et al.* (2015) from short read alignments. Of the 129 SNPs called here, 37 were polymorphic amongst the samples, in contrast to 78 in the Darch *et al.* (2015) data. Tables S1–S3 list the totals of the different classes of variants and the effects of variant filters (see Data Bibliography items 8 and 9 for tables containing each variant). The congruity between SNPs called in this analysis and those reported by Darch *et al.* (2015) is low (Fig. 2), although 41 within-sample SNPs in the latter were called as fixed here. Several variants were called in both studies but subsequently filtered by our novel algorithms. Most were adjacent to prophage in the reference that were missing in the samples or at repetitive regions associated with paired reads mapping with unexpected separation distances (Fig. 3). Additionally, the previous study reported an absence of insertions or deletions causing frameshifts in protein-coding regions either amongst, or compared with the reference sequence, which is atypical for *P. aeruginosa* isolated from chronic CF airway infections (Rau *et al.*, 2010; Marvig *et al.*, 2013). In this analysis, eight protein-coding regions were polymorphic for frameshift indels amongst the Nottingham isolates: PLES_RS00075, PLES_RS001530, PLES_RS05000 (*mpl*), PLES_RS06070, PLES_RS12750, PLES_RS15875 (*pslJ*), PLES_RS18160 (*mltD*), PLES_RS18915 (*stk1*). A further six were fixed relative to the reference genome (LESB58): PLES_RS02170 (*mexB*), PLES_RS16380, PLES_RS25200 (*ampD*), PLES_RS27535, PLES_RS28165 and PLES_RS30445 (Table S4). In total, 37 indels were inferred in the Nottingham isolates relative to LESB58, 22 of which were polymorphic amongst them.

The overall higher number of variants, particularly indels, reported in the present analysis might reflect improved sensitivity of the newer GATK HaploTypeCaller module (McKenna *et al.*, 2010) over the GATK Unified Genotype caller used by Williams *et al.* (2015) and SAMTools (Li *et al.*, 2009) used by Darch *et al.* (2015). This characteristic has been demonstrated in previous benchmarking reports (Chapman, 2014). The joint genotyping performed in the GATK-based analyses, where information across samples is combined, might also have contributed to the incongruence with the Darch *et al.* (2015) results.

**Fig. 4.**
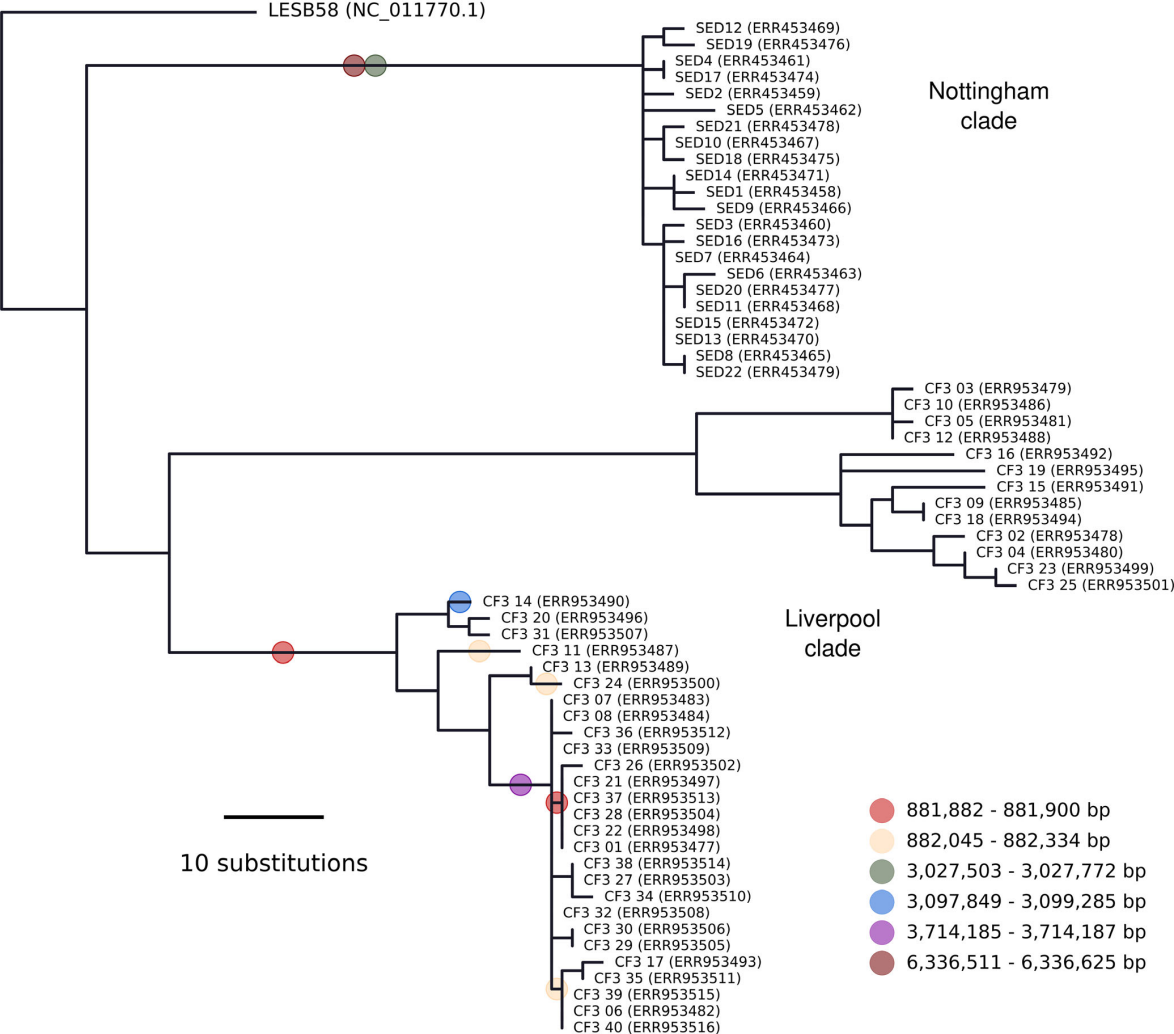
Maximum-likelihood phylogenetic reconstruction for 63 *P. aeruginosa* LES isolates. All were isolated from CF patient sputa: 40 were isolated from a single sample obtained at a clinic in Liverpool, UK in 2009 (ERR953477–ERR953516) and 22 were isolated from a single sample obtained at a clinic in Nottingham, UK in approximately 2014 (ERR453458–ERR453479). The remaining outgroup isolate, LESB58, was isolated in 1988 in Liverpool, UK. The coloured circles indicate branches that imported DNA by homologous recombination as inferred by ClonalFrameML. The positions in the key correspond to the LESB58 genome sequence.

Phylogenetic reconstruction (Fig. 4) recovered the two distinct lineages in Liverpool patient CF3 reported previously (Williams *et al.*, 2015) and a single lineage of lower diversity from the Nottingham data. Despite some differences in bioinformatics (Fig. 1), the topology within each Liverpool lineage was similar to that reported previously (Williams *et al.*, 2015) with an unweighted Robinson–Foulds distance of 44 between trees with 79 bipartitions (Robinson & Foulds, 1981). The topology within the 22-isolate Nottingham lineage was entirely incongruent to that reported by Darch *et al.* (2015): these trees with 43 and 41 bipartitions each differed by a distance of 42. This metric is the sum of the edges present in one tree but not the other and vice versa. In the latter case, 42 is the maximum distance given the number of tips and resolution of each topology i.e. the topologies could not be more incongruent.

Recombination was inferred using BRAT NextGen (Marttinen *et al.*, 2012) and, for importations only, ClonalFrameML (Didelot & Wilson, 2015). Amongst the 22 Nottingham genomes analysed together, BRAT NextGen detected a single region from 433 989 to 2 386 565 bp imported by one isolate (SED5). ClonalFrameML did not detect any imported regions. BRAT NextGen analysis of the combined Liverpool and Nottingham data yielded nine distinct origins of foreign genomic segments amongst the 62 sequences, with a single region of common origin amongst all 22 Nottingham isolates. ClonalFrameML reported six haplotypes (distinct chromosome regions), totalling 2134 bp with eight individuals, extant or ancestral, affected. These are indicated in Fig. 4 and have only two regions, close to 0.8 Mb in the larger Liverpool clade, that are in partial agreement with the BRAT NextGen analysis. Thus, the present analysis indicates the Liverpool and Nottingham data sharing similarly low rates of recombination, and does not corroborate the high rates of homologous recombination for *P. aeruginosa* in chronic CF lung infections reported by Darch *et al.* (2015).

### Potential causes of incongruity between studies

Recombination detection sensitivity decreases with diversity (Marttinen *et al.*, 2012); thus, the lower recombination rate inferred here can be partly explained by the fewer variants found in this study than in Darch *et al.* (2015): 37 versus 78 SNPs. However, in addition to read mapping, multiple sequence alignment of short read *de novo* assemblies of the 22 genome sequences yielded a nearly 40-fold larger set of 1436 SNPs in the Darch *et al*. (2015) study (Fig S2). This high-diversity SNP collection was used for BRAT NextGen analysis, indicating many more historical recombination events. Multiple sequence alignment of *de novo* assemblies is a more complex problem to solve than short read mapping. With more complexity, errors in each of the assemblies might have compounded errors of multiple alignment, i.e. false-positive SNPs. Alternatively, the short read mapping approaches might have missed many true-positive variants, including those in variably present chromosome regions that cannot be aligned to the LESB58 reference sequence. We quantified missing regions by *de novo* assembly of reads that did not align or aligned poorly for each isolate genome: on average 38 kb amongst 25 contigs. Thus, the read mapping approach missed 0.57 % of each genome, consistent with a pan-genome analysis by Darch *et al.* (2015) that indicated only three genes missing in the reference and present in more than one isolate. Furthermore, their BRAT NextGen analysis indicated recombinant regions along the whole genome length. Thus, it seems most variants are likely to be in regions tested by the read mapping approaches.

We also performed a series of benchmarking and validation tests on our variant calling approach. In simulated reads from LESB58 genome sequences with known variants added *in silico*, all SNPs were called correctly with no false positives (Table 1). Darch *et al.* (2015) reported no indels, whereas the present analysis found several. Across our 10 simulations, 295 out of 300 indels were correct within 2 bp. In simulations 5–10, large deletions were added to simulate absence of LESB58 Genomic Island 5 and prophage 5. Polymorphic false-positive insertions were called by GATK, but correctly filtered by our novel ‘rearrangements’ filter.

**Table 1.** Accuracy of the variant calling pipeline for SNPs and indels

Simulation no.	Total SNPs	True-positive SNPs	False-positive SNPs	False-negative SNPs	Total indels	True-positive indels	False-positive indels*	False-negative indels
1	51	51	0	0	30	24	6	6
2	48	48	0	0	30	20	10	10
3	47	47	0	0	30	24	6	6
4	52	52	0	0	30	23	7	7
5	50	50	0	0	30	22	8	8
6	47	47	0	0	30	21	9	9
7	52	52	0	0	30	21	9	9
8	53	53	0	0	30	21	9	9
9	51	51	0	0	30	20	11	10
10	50	50	0	0	30	22	14	8

* All but five false-positive indels are within 2 bp of the correct call.

We validated each SNP by performing *de novo* assemblies of the reads aligned to the same region and checking the resulting contigs for the variant. Our novel ‘rearrangements’ and ‘repeats’ filters improved variant corroboration from 88–98 to 97–100 %; thus, they apparently omitted false-positive SNPs called in short read alignments but absent in the contigs (Table S5). Amongst variants removed by the ‘rearrangements’ filter were seven SNPs and three indels either side of LESB58 prophage 5 – a genomic feature absent in the Nottingham isolates. To test whether the filter was performing correctly in this instance, we repeated the variant calling, but used LESlike7 instead – an alternative reference genome without this prophage. No polymorphisms were called around the region in LESlike7 orthologous to the prophage insertion site, thus validating that the filter had removed false positives. The other novel filter was designed to detect repeat regions in the reference genome longer than the sequencing fragment length, which is known to cause ambiguity in mapping reads (Olson *et al.*, 2015). Repeats identified included a previously documented repeat within prophage 2 at 0.895-0.899 Mb and prophage 3 at 1.465-1.469 Mb (Winstanley *et al.*, 2009). Nineteen SNPs were filtered from this region that also spanned numerous SNPs inferred as adaptive and recombinant by Darch *et al.* (2015). Notably, the divergence between these repeat units was enough to confuse the read aligner: the alignment contained double the mean read depth over one region, but very few reads at the other (Fig. 3). Furthermore, the ‘rearrangements’ filter detected the edges of the repeat units, between which read pair members were arbitrarily aligned, causing unexpected mapping distances. Both filters are therefore omitting confirmed false positives or variants at repeats indicated as unreliable because of improper paired-read mapping. The filtering of true positives, causing underestimation of diversity and potentially of recombination, remains a possibility. However, the benchmarking and validation suggest this effect is small.

## Conclusion

Our analysis suggests homologous recombination has made a minor contribution to *P. aeruginosa* LES diversity in two chronic CF airway infections compared with spontaneous mutations. Within-host adaptive evolution might therefore be limited by the mutation rate, rather than the effects of homologous recombination, potentially explaining the prevalence of hypermutator isolates in CF clinical samples (Oliver *et al.*, 2000) including LES (Kenna *et al.*, 2007). A recent study of *P. aeruginosa* diversity in different regions of chronically infected CF lungs suggests that there exists strong spatial separation of subpopulations within the lung (Jorth *et al.*, 2015). Thus, genetically diverged lineages, even if coexisting within the same lung, may not encounter each other often enough to exchange DNA at detectable levels. The novel variant filters allow data-driven exclusion of probable false positives with minimal intervention from the researcher. The rearrangements filter, designed to mitigate false positives caused by missing chromosome regions, is of particular value given the abundance and rapid dynamics of prophage and genomic islands in microbial communities (Zhou *et al.*, 2011).

## Supplementary Data

888 Supplementary Data
