# Supplementary material for: Refined analyses suggest that recombination is a minor source of genomic diversity in Pseudomonas aeruginosa chronic cystic fibrosis infections
Source: Microb Genom. 2016 Mar 2;2(3):e000051. doi: 10.1099/mgen.0.000051 (PMC5320575; doi:10.1099/mgen.0.000051)
Supplement: Supplementary file 1 — Supplementary Data [file mgen-02-51-s001.pdf]

Supplementary Materials for:

Refined analyses suggest recombination is a minor source of genomic diversity in *Pseudomonas aeruginosa* chronic cystic fibrosis infections.

METHODS

Short Read Preparation

Preprocessing of short read data was implemented in the PrepareReads module of BAGA. Large FASTQ read files were subsampled to provide a 80x read coverage for mapping to a 6.6 Mb genome. Reads were trimmed using position-specific quality scores with Sickle ver. 1.33 (Joshi & Fass 2011) . Adaptor sequences from library preparation were removed using Cutadapt ver. 1.9.dev0 (Martin 2011) .

Short Read Alignment to reference genome

Short read alignment was implemented in the AlignReads module of BAGA. Paired end short reads were aligned to the LESB58 reference genome sequences with the MEM algorithm of BWA (Li & Durbin 2009) (github.com repository revision 9812521) estimating insert size from the data for designation of "proper pair" reads. SAMTools (github.com repository revision b0525f3) (Li et al. 2009) was used for conversion of SAM to BAM files and BAM file sorting. Duplicate reads were removed from alignments using Picard version1.135. The IndelRealigner module of the Genome Analysis Toolkit (GATK) (McKenna et al. 2010; DePristo et al. 2011) was used to simultaneously re-align reads that were likely to be near insertions or deletions.

Reference genome repetitive region variant filter

The algorithm for identifying reference genome repeat regions in which variants were deemed unreliable and omitted was implemented in the Repeats module of BAGA. First, each open reading frame nucleotide sequence in the published annotation was aligned back to the full LESB58 chromosome sequence using BWA MEM lowering alignment stringency by setting mismatch penalty to 2 and gap open penalty to 3. These initial alignments using BWA are used to seed an alignment procedure that uses optimal global (Needleman-Wunsch) alignment for better accuracy. The lower alignment stringency produces alignments more divergent than the target 98% nucleotide identity. After omitting alignments between the same sequences, the resulting collection of ORFs within

repeat regions were organised into contiguous blocks of homologous sequence. Each pair of homologous sequences, which may now contain more than one pair of ORFs, were aligned using the Needleman-Wunsch optimal global alignment algorithm implemented in the seq-align package (github.com/noporpoise/seq-align revision 0589383). The percent identity between the two aligned regions is calculated within each 100 bp window at 20 bp increments along a pairwise alignment and regions with more than 98% nucleotide sequence identity were deemed similar enough to yield ambiguous read alignments. Regions greater than the mean insert size are retained for filtering because smaller regions are expected to be resolved by the relative positions of paired-end reads.

Genome rearrangements variant filter

This algorithm was implemented in the Structure module of BAGA and is applied on a per sample basis. The threshold for the ratio of reads not in a proper pair, to those in a proper pair, which when exceeded defines regions in which variant calls should be omitted, was set to 0.15. At this threshold, false positive variants in the simulations where large deletions were addedwere successfully filtered out. This ratio, collected at each position in the reference genome, is “smoothed” by averaging over a 500 bp moving window. Proper pair status was determined by the BWA MEM aligner and was acquired from BAM files using PySAM version 0.8.3. Each region for variant omission was extended in each direction if a moving window equal to the mean insert size had at least 15% without any aligned reads. Plotting is implemented in the Structure module.

Variant calling

Variant calling was implemented in the CallVariants module of BAGA which follows the “Best Practices workflow” provided by the developers of GATK (Van der Auwera et al. 2013) (https://www.broadinstitute.org/gatk/guide/best-practices accessed 15/04/2015). SNPs and indels were called simultaneously via local re-assembly of haplotypes using the HaplotypeCaller module of the GATK setting –sample_ploidy to 1, –heterozygosity to 0.0001 and –indel_heterozygosity to 0.00001 to approximate the 100-1400 SNPs in the 6.6Mb genomes reported by Williams et al. (2015) and Darch et al. (2015) and the ten-fold few indels reported by Williams et al. (2015). The two datasets were called in separate joint analyses. The standard GATK hard filters for SNPs and indels were applied. Base quality scores in BAM files were recalibrated.

Population structure analysis

The following procedures were implemented in the ComparativeAnalysis module of the BAGA. A multiple sequence alignment including the reference genome and all isolates was generated from the called SNPs with all filters applied. Invariant positions were retained and columns with missing data were included except if missing in all samples. For example, all 22 Nottingham samples are missing LESB58 Genomic Island 5 which is 50 kb long. A pair of polymorphisms 50 bases either side of the insertion site of Genomic Island 5 should be considered 100 bases apart in the 22 sampled genomes, not 50,100 bases apart, as it is only in the reference genome. Maximum likelihood phylogenetic reconstruction was performed using PhyML (github.com/stephaneguindon/phyml revision e71c553) (Guindon et al. 2010) using the default settings. Recombination was inferred using ClonalFrameML

(github.com/xavierdidelot/ClonalFrameML revision 2d793a3) (Didelot & Wilson 2015) using the PhyML phylogeny and -ignore_incomplete_sites true. Plotting including mapping the alignment positions reported by ClonalFrameML back to the reference genome is implemented in the ComparativeAnalysis module of BAGA. Phylogeny manipulations were performed using DendroPy 4.0.3 (Sukumaran & Holder 2010). Further detection of recombination events, not implemented in BAGA, was performed using BRAT NextGen version 4/18/2011(Marttinen et al. 2012) with 100 iterations to achieve negligible changes in model parameter estimates over the last 50 iterations. A threshold of 5% applied to 100 permutations was used for significance testing of each event.

In silico read generation

Ten sets of paired reads were generated from the LESB58 reference sequence using GemSim version 1.6 (McElroy et al. 2012) and modelling the error profile of the Illumina GA IIx with Illumina Sequencing Kit v4 chemistry. Read length was 100 bp, with insert size 350 bp and standard deviation 20. To provide 60x coverage depth, 1,980,527 read pairs were generated. Each set of reads was generated from a reference sequence on which 50 SNPs (30 shared with ~20 additionally selected at random to approximate a tree-like distribution) and 15 insertions and deletions (five shared, 10 genome specific) had been applied *in silico*. These simulated reads can be reproduced using the SimulateReads module of BAGA and the scripts provided in the figshare repository(http://dx.doi.org/10.6084/m9.figshare.2056365). The read data was then analysed with the BAGA pipeline described above.

Region-specific de novo assembly of reads for SNP validation

For each isolate read set, at each SNP position, reads mapping to 5000 bp each side were extracted from BAM files using pySAM. These were combined with the unmapped and poorly mapped reads for de novo assembly using SPAdes. By reducing the total reads per assembly we aimed to decrease assembly complexity and increase accuracy. A set of contigs were also assembled from just the unmapped and poorly mapped reads and subtracted from the contigs of each SNP-associated assembly to leave those contigs relevant to that region. These remaining contigs were pairwise aligned to the region using the optimal global Needleman-Wunsch algorithm implemented in Seqalign. The pairwise alignment was then check for the presence or absence of the SNP.

RESULTS AND DISCUSSION

Impact of reference genome repeats on variant calling

In the present analysis, two novel filters were applied to mitigate false positive errors. For each filter, variants were omitted if called in regions where assumptions of the method were likely to be violated: either within long repeats in the reference sequence, or near break-points at rearrangements between a sample and the reference. Calling variants by mapping whole-genome shotgun reads to a reference genome assumes each gene sequence in the sampled individual must have a unique

equivalent in the reference. This 1-to-1 relationship means that each read will align unambiguously to the position in the reference genome that is positionally orthologous to the read origin in the sampled genome. If a read is aligned to the wrong (non-orthologous) part of a reference genome, false positive variants calls are likely.

The first novel variant filter in this analysis omitted variants in long repeats in the reference sequence. Sequence repeats can be caused by duplications (paralogy) or horizontally acquired DNA incorporated into a chromosome whilst also being homologous to another part of the chromosome. The Burrow-Wheeler Aligner used in this analysis reports an alignment quality of zero where a read maps perfectly to two parts of a genome. In these cases, the risk of false positive variants is easily mitigated by excluding reads with an alignment quality of zero. However, these zero alignment qualities will not be reported for slightly divergent repeats, which would be the case when one or more substitutions have occurred at one repeat unit but not another.

The algorithm we developed to identify repeat regions was implemented in the Repeats module of the Bacterial and Archaeal Genome Analyser (BAGA). Of the 6.6 Mb LESB58 reference genome, 75.1 kb of chromosome in 35 regions were deemed repetitive. These regions required at least 98% aligned nucleotide identity and a length greater than the mean sequencing library insert size because smaller regions are expected to be resolved by the relative positions of paired-end reads. Only contiguous repeat regions, tandem or otherwise, longer than the insert size used in library preparation were included in the filter because shorter regions should be resolvable as unique. Of the 42.7 kb in previously described long duplications in LESB58 (Winstanley et al. 2009), 23.0 kb were included in the high identity regions reported here. Among the Nottingham data, eight SNPs within these regions were omitted (Table S2). In the Liverpool data, nine SNPs were omitted. No indels were found in these regions.

Impact of genome sequence rearrangements on variant calling

The second novel variant filter used in this analysis omitted variants in regions around break-points of chromosomal rearrangements. The assumed 1-to-1 orthology required for variant calls from aligned reads can be violated at the regions bordering rearrangements between a sample and the reference genome. These structural changes in bacteria include large deletions, inversions, duplications and integrations, the last typically caused by acquisition of prophage or genomic islands. Some phage are known to carry a fragments of tRNA genes with homology to those in the host organism and integration involves replacement of parts the host version of a tRNA gene with the phage version (Campbell 1992) . Thus, regions flanking the resulting prophage may have sequence divergence caused by a more complex history than the simple orthology assumed by the read mapping method with an increased risk of artefactual, false-positive variants.

The algorithm we developed for omitting putatively unreliable variants near regions of structural rearrangements was implemented in the Structure module of the BAGA software package. The algorithm exploits the fact that sequence reads in this analysis were “paired-end”: from either end of many template DNA fragments of similar lengths. The Burrows-Wheeler Aligner used in this analysis assigns a read “proper pair” status if the aligned distance and orientation to its paired read is close to that expected given the mean template DNA fragment length. Paired-end reads on either side of a rearrangement break-point typically align to the reference at distances far greater than the fragment lengths or in different orientations. For example, in many samples, no reads mapped to the LESB58 chromosome region annotated as “LES prophage 5” indicating absence of the prophage in those samples. Paired reads would align kilobases apart on either side of the prophage if they originated from a DNA fragment spanning the orthologous site of prophage integration, in a sample lacking the prophage. These reads would not be assigned “proper pair” status being so much further apart than the mean fragment size.

Fig. 3 illustrates how our algorithm determines regions near genome rearrangements in which variant calls may be unreliable. In this example the rearrangement is a duplication, but other common rearrangements include the loss or gain of a prophage or other genomic island. These regions are defined by a deviation in the ratio of proper-pair-assigned reads to non-proper-pair-assigned reads: the more non-proper-pair-assigned reads, the less reliable the region. The initially selected region is extended while regions of the reference have zero aligned read coverage and variants called within the combined region are omitted. In the Nottingham data, 33 SNPs and 10 indels within these regions were omitted (Table S2). In the Liverpool data, 39 SNPs and eight indels were omitted. This analytic approach, by accounting for potential false positive variant calls caused by missing regions of chromosome, is of value because turnover of prophage and genomic islands among microbial genomes is often rapid(Wilmes et al. 2009). In many cases, including the analyses of the Liverpool and Nottingham datasets, guaranteeing a similar genomic composition to permit a contiguous alignment between reference and sampled genomes is difficult. Prophage and genomic islands and are abundant throughout Bacteria and Archaea(Zhou et al. 2011)so few read mapping analyses will be unaffected by these challenges.

TABLES

Table S1: Small insertions and deletions predicted to cause frame-shift mutations among the Nottingham isolates

Chromosome	Position (bp)	Reference	Variant	Gene ID	Gene name	Frequency	Frequency (filtered)
NC_011770.1	14914	T	TGC	PLES_RS00075		10	10
NC_011770.1	335247	C	CG	PLES_RS01530		2	2
NC_011770.1	467972	GATCCTCCTCGT	G	PLES_RS02170	mexB	22	22
NC_011770.1	1037925	A	AC	PLES_RS05000	mpl	3	3
NC_011770.1	1267074	CCCGCTGGAGCT	C	PLES_RS06070		3	3
NC_011770.1	2532081	G	GT	PLES_RS12255		1	0
NC_011770.1	2639073	AG	A	PLES_RS12750		1	1
NC_011770.1	3280614	A	AAGCGCGCGACCCGGAAGCCGTTGCCCAGCGGGGCGCCCTGT	PLES_RS15460		1	0
NC_011770.1	3378367	AG	A	PLES_RS15875	pslJ	21	21
NC_011770.1	3388596	GTACTTGCCGCTGTCCAGGTAGGACTGGGCGGTGGCCTGGTCGGGTTTCT	G	PLES_RS15915	pslB	1	0
NC_011770.1	3484676	CGCCCGAGCAA	C	PLES_RS16380		22	22
NC_011770.1	3898444	C	CACGATTCGTTGTCAAAAATAGCCAAGGACCCGGACACACGCCTGATGCG	PLES_RS18160	mltD	1	1
NC_011770.1	4044433	C	CGG	PLES_RS18915	stk1	1	1

NC_011770.1	4931526	G	GGGGATGTCGAC ATGCAA	PLES_RS23105		2	0
NC_011770.1	5398560	TCG	T	PLES_RS25200	ampD	22	22
NC_011770.1	5903286	GCT	G	PLES_RS27535		22	22
NC_011770.1	6060546	C	CAT	PLES_RS28165		22	22
NC_011770.1	6557920	GGGGCGGCAGCA GTGGCGTTCGGC GCAGT	G	PLES_RS30445		22	22

Table S2: Total polymorphisms called for the Liverpool and Nottingham datasets as cumulative filters are applied, divided by type

	Liverpool		Nottingham		All data	
Cumulative filters applied	SNPs*	Indels†	SNPs	Indels	SNPs	Indels
None	318	68	170	47	440	89
GATK‡ standard 'hard' filter	318	68	170	47	440	89
BAGA§ reference genome repeats	309	68	162	47	424	89
BAGA genome rearrangements	270	60	129	37	364	78

*Single nucleotide polymorphisms; †Small insertion or deletion polymorphisms; ‡Genome Analysis Toolkit; §Bacterial and Archaeal Genome Analyser

Table S3: Polymorphisms called as fixed in samples and differing with reference for the Liverpool and Nottingham datasets as cumulative filters are applied, divided by type

	Liverpool		Nottingham		All data	
Cumulative filters applied	SNPs*	Indels†	SNPs	Indels	SNPs	Indels
None	43	5	101	17	113	21
GATK‡ standard 'hard' filter	43	5	101	17	113	21
BAGA§ reference genome repeats	43	5	99	17	111	21

BAGA genome rearrangements	43	5	92	15	104	19

*Single nucleotide polymorphisms; †Small insertion or deletion polymorphisms; ‡Genome Analysis Toolkit; §Bacterial and Archaeal Genome Analyser

Table S4: Polymorphisms called among the Liverpool and Nottingham datasets as cumulative filters are applied, divided by type

	Liverpool		Nottingham		All data	
Cumulative filters applied	SNPs[*]	Indels[†]	SNPs	Indels	SNPs	Indels
None	275	63	69	30	337	71
GATK[‡] standard 'hard' filter	275	63	69	30	337	71
BAGA[§] reference genome repeats	266	63	63	30	322	71
BAGA genome rearrangements	227	55	37	22	264	60

*Single nucleotide polymorphisms; †Small insertion or deletion polymorphisms; ‡Genome Analysis Toolkit; §Bacterial and Archaeal Genome Analyser

Table S5: Proportion of single nucleotide polymorphisms called by the GATK Haplotype Caller recovered in de novo assembled contigs.

Sample	Proportion all variants corroborated	Proportion filtered variants corroborated*
SED01	96.83%	100.00%
SED02	92.56%	96.00%
SED03	97.54%	100.00%
SED04	95.97%	100.00%
SED05	92.48%	98.04%
SED06	94.92%	100.00%
SED07	91.41%	97.94%
SED08	93.75%	97.98%
SED09	92.48%	99.02%

SED10	94.26%	97.96%
SED11	89.84%	97.96%
SED12	93.10%	96.94%
SED13	88.10%	100.00%
SED14	94.40%	100.00%
SED15	91.41%	97.96%
SED16	93.75%	99.00%
SED17	94.49%	100.00%
SED18	95.31%	100.00%
SED19	92.91%	97.98%
SED20	92.11%	96.94%
SED21	95.97%	98.98%
SED22	96.09%	100.00%

203 *Most of the variants removed by the novel filters were not present in the *de novo* assemblies

FIGURES

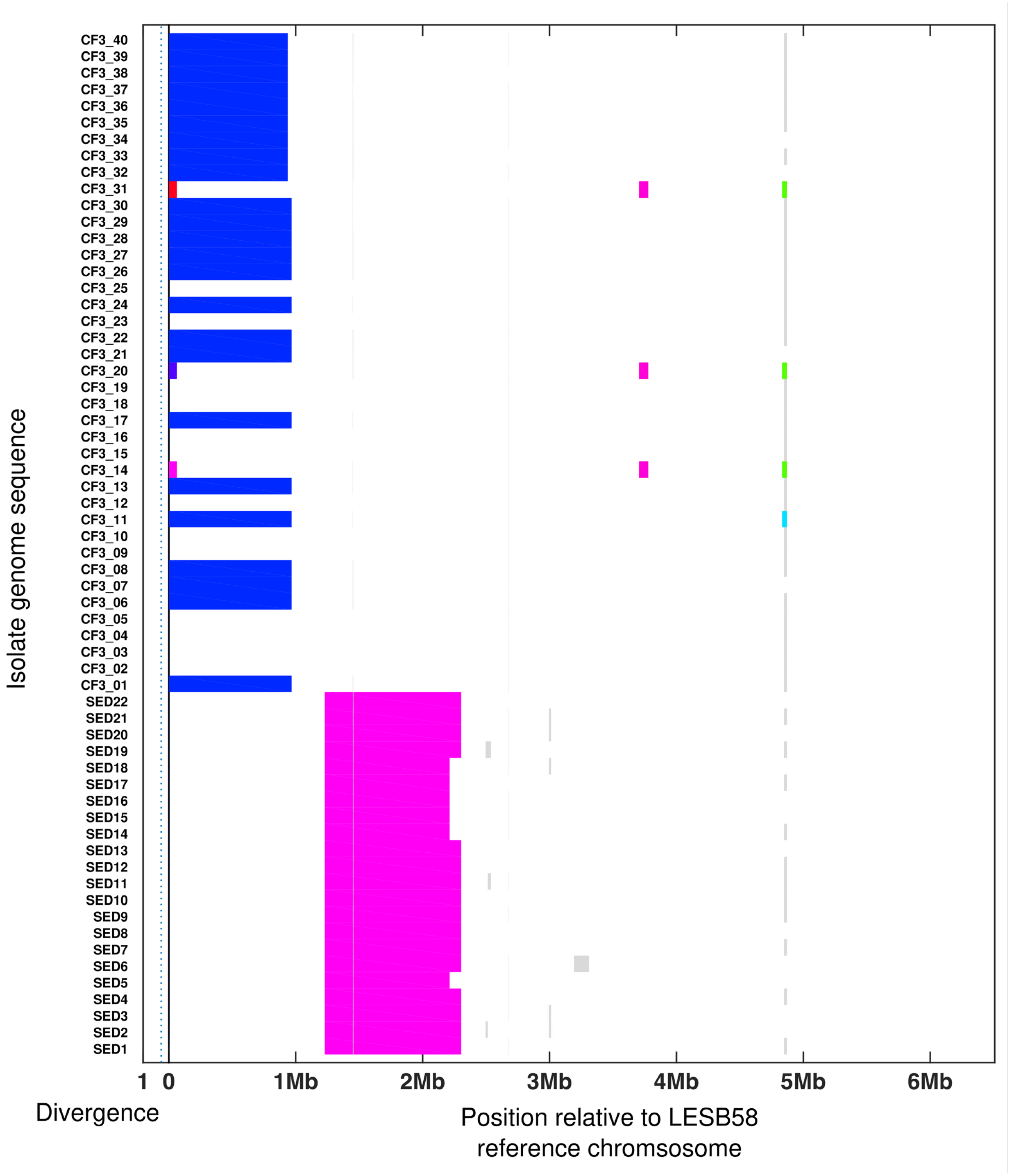

Figure S1. Segments of chromosome indicated by coloured bars, among the Liverpool and Nottingham isolates, inferred to have undergone homologous recombination. Regions that are the same colour

share a common originwhereas grey regions were not deemed significant in a permutation test at alpha = 0.05.

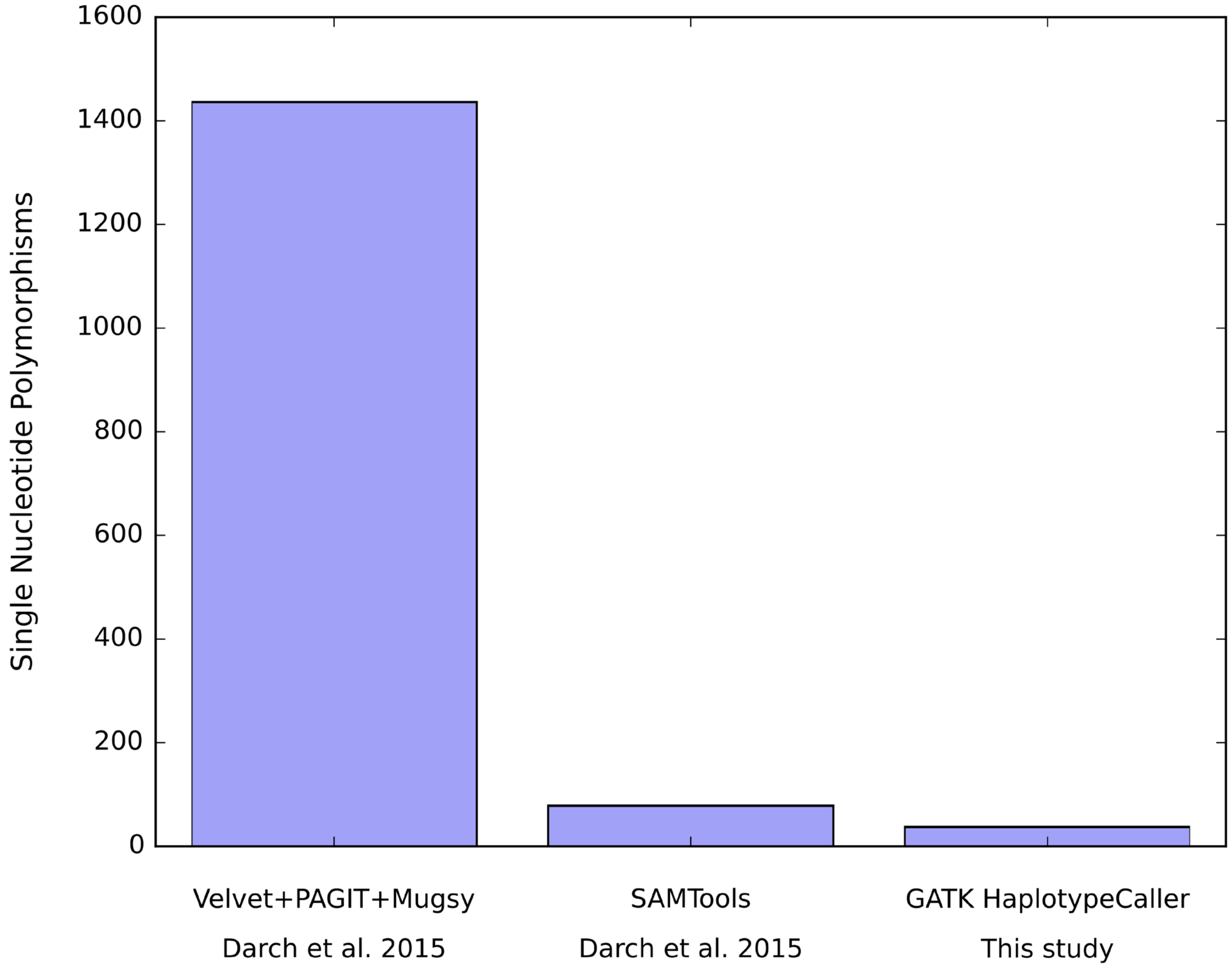

Figure S2. Total single nucleotide polymorphisms called by different methods from the same dataset. In the first method from a previously published analysis, short reads from 22 *P. aeruginosa* isolates were each assembled into contigs *de novo*, improved using an automated pipeline and finally combined into a multiple sequence alignment within which polymorphismic positions were counted. In the second two methods one from the previous study and the third from this report, variants were called among short reads aligned against a complete reference sequence from a closely related isolate (*P. aeruginosa* LESB58).

REFERENCES

Sickle: A sliding-window, adaptive, quality-based trimming tool for FastQ files (Version 1.29) [https://github.com/najoshi/sickle]

Martin M. (2011). Cutadapt removes adapter sequences from high-throughput sequencing reads. *EMBnetjournal***17** 1.

Li H., Durbin R. (2009). Fast and accurate short read alignment with Burrows–Wheeler transform. *Bioinformatics***25** 1754-1760.

Li H., Handsaker B., Wysoker A., Fennell T., Ruan J., Homer N., Marth G., Abecasis G., Durbin R. (2009). The Sequence Alignment/Map format and SAMtools. *Bioinformatics***25** 2078-2079.

Picard Sequence Alignment/Map file manipulation library [http://picard.sourceforge.net/]

McKenna A., Hanna M., Banks E., Sivachenko A., Cibulskis K., Kernytsky A., Garimella K., Altshuler D., Gabriel S., other authors (2010). The Genome Analysis Toolkit: A MapReduce framework for analyzing next-generation DNA sequencing data. *Genome Res***20** 1297-1303.

DePristo M.A., Banks E., Poplin R., Garimella K.V., Maguire J.R., Hartl C., Philippakis A.A., del Angel G., Rivas M.A., other authors (2011). A framework for variation discovery and genotyping using next-generation DNA sequencing data. *Nat Genet***43** 491-498.

Van der Auwera G.A., Carneiro M.O., Hartl C., Poplin R., del Angel G., Levy-Moonshine A., Jordan T., Shakir K., Roazen D., other authors (2013). From FastQ Data to High-Confidence Variant Calls: The Genome Analysis Toolkit Best Practices Pipeline. *Curr Protoc Bioinform***43** 11.10.1-11.10.33.

Guindon S., Dufayard J., Lefort V., Anisimova M., Hordijk W., O. G. (2010). New algorithms and methods to estimate maximum-likelihood phylogenies: assessing the performance of PhyML 3.0. *Syst Biol***59** 307-21.

Didelot X., Wilson D.J. (2015). ClonalFrameML: Efficient Inference of Recombination in Whole Bacterial Genomes. *PLoS Comput Biol***11** e1004041-.

Sukumaran J., Holder M.T. (2010). DendroPy: a Python library for phylogenetic computing. *Bioinformatics***26** 1569-1571.

McElroy K.E., Luciani F., Thomas T. (2012). GemSIM: general, error-model based simulator of next-generation sequencing data. *BMC Genomics***13** 74.

Winstanley C., Langille M.G., Fothergill J.L., Kukavica-Ibrulj I., Paradis-Bleau C., Sanschagrin F., Thomson N.R., Winsor G.L., Quail M.A., other authors (2009). Newly introduced genomic prophage islands are critical determinants of *in vivo* competitiveness in the Liverpool Epidemic Strain of *Pseudomonas aeruginosa*. *Genome Res***19** 12-23.

Campbell A.M. (1992). Chromosomal insertion sites for phages and plasmids. *J Bacteriol***174** 7495-7499.

Wilmes P., Simmons S.L., Denef V.J., Banfield J.F. (2009). The dynamic genetic repertoire of microbial communities. *FEMS Microbiol Rev***33** 109-132.

Zhou Y., Liang Y., Lynch K.H., Dennis J.J., Wishart D.S. (2011). PHAST: A Fast Phage Search Tool. *Nucleic Acids Res***39** W347-W352.
